# The Causes and Prevention of Suicide

**Published:** 1884-03

**Authors:** 


### The Causes and Prevention of Suicide.

That there is a steady increase of suicide in all civilized lands, seems acknowledged by the best statisticians. In a review of Prof. Reclam's writings on the subject, a German critic agrees that one fertile cause is the over-education of the masses—giving young people tastes and ambitions which in the vast majority of cases cannot be gratified, and thus rendering them discontented, and at last desperate. Another cause is the growing belief that material success is alone worth an effort—in other words, the neglect of spiritual religion and elevated philosophy. A third is the excessive use of distilled spirits; and, a fourth, the oppression of a forced military service.

